# Supplementary material for: Selective Retrieval of Individual Cells from Microfluidic Arrays Combining Dielectrophoretic Force and Directed Hydrodynamic Flow
Source: Micromachines (Basel). 2020 Mar 20;11(3):322. doi: 10.3390/mi11030322 (PMC7143322; doi:10.3390/mi11030322)
Supplement: Supplementary file 1 [file micromachines-11-00322-s001.zip › Supplementary_Materials.docx]

Supplementary Materials

Selective Retrieval of Individual Cells from Microfluidic Arrays Combining Dielectrophoretic Force and Directed Hydrodynamic Flow

Pierre-Emmanuel Thiriet *, Joern Pezoldt, Gabriele Gambardella, Kevin Keim, Bart Deplancke and Carlotta Guiducci


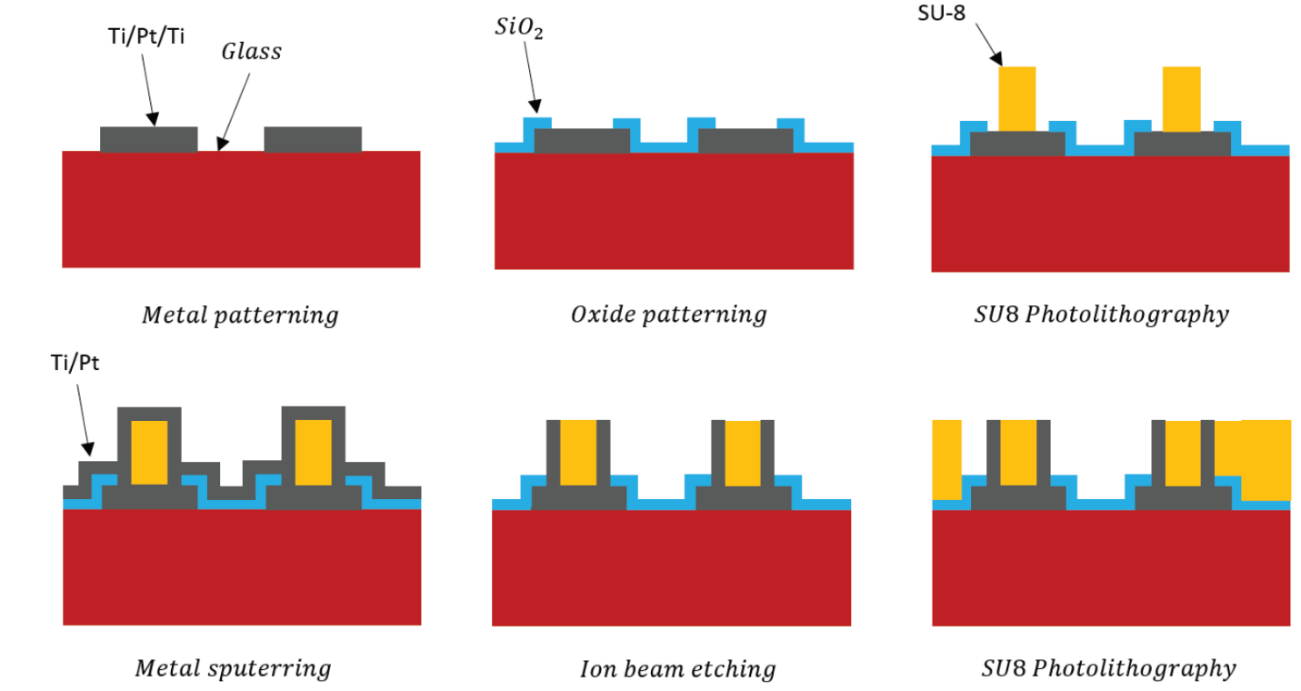


**Figure S1: Microfabrication process flow.** Fabrication of vertical electrodes extruding from microfluidic channels. Schematic view of the microfabrication process. First, planar metal lines are partially insulated by silicon di-oxide and successively 15 µm-high SU-8 pillars are deposited on top of the open metal regions. Those pillars are then covered with a metal layer that is then removed everywhere but on the pillars walls through vertical etching. Electrical connection between the vertical metal layers on the pillars and the horizontal lines is maintained.


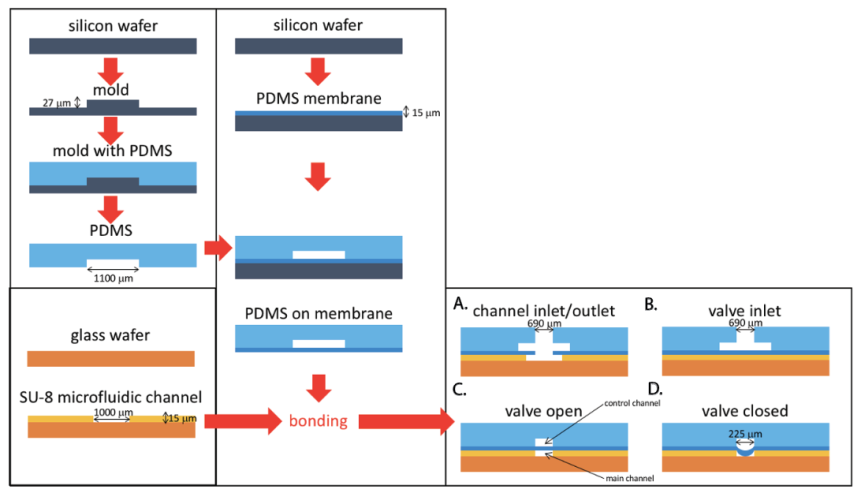


**Figure S2. Valve fabrication, Fabrication of PDMS coverslips**. Gray-blue: silicon wafer; marine blue: PDMS membrane; sky blue: thick PDMS; orange: glass wafer; yellow: SU-8. The membrane, obtained by spin-coating on a blank silicon wafer, is bonded to the control PDMS layer, previously punched at the entrance of the control channels. The PDMS coverslip is then punched at the entrance of the microfluidic main channels and bonded to the SU-8 microfluidic channel. Right inset: four cross-section views are provided to better illustrate the operation of the device: channel inlets/outlets with access to the main channel (configuration A); valve inlets with access to the control channel (configuration B); valves above the channel, either open (no pressure, configuration C) or closed (pressure in the control channel, configuration D).

| 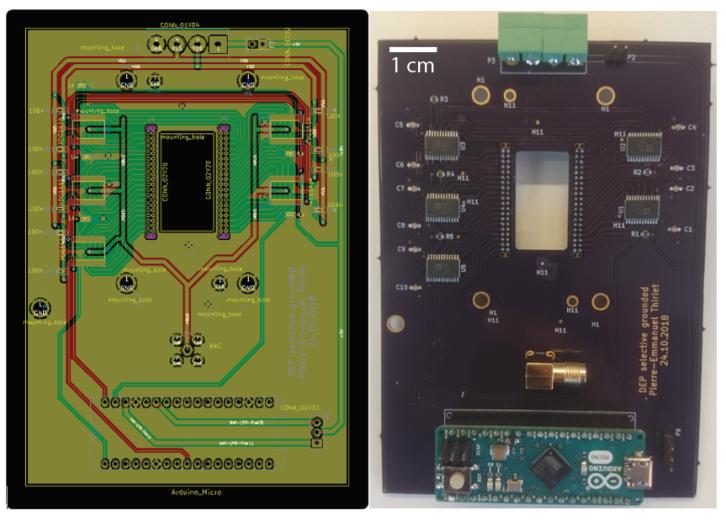 | |
| --- | --- |
| **(a-A)** | **(a-B)** |
| 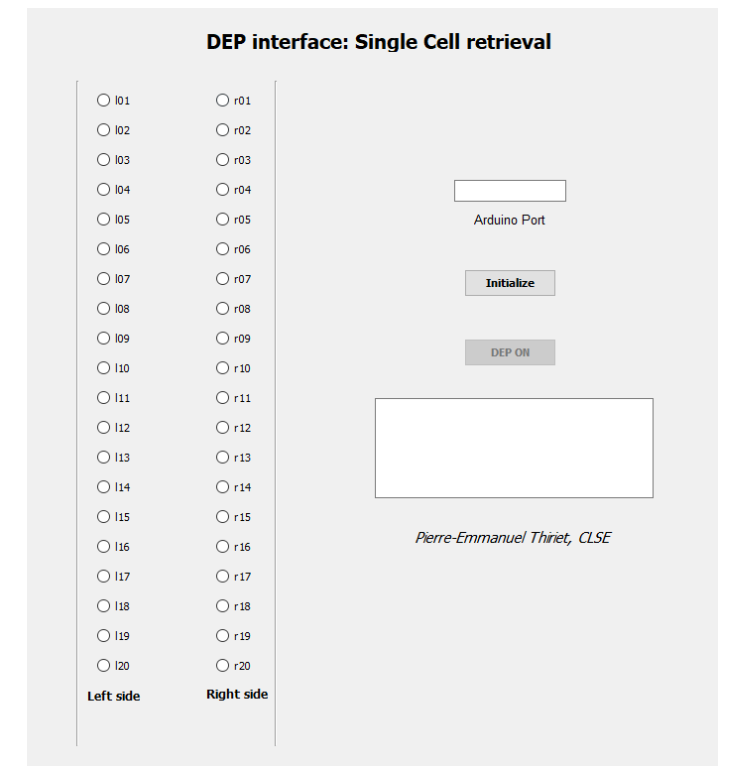 | |
| **(b)** | |

**Figure S3: PCB design and operation.** In order to carry out selective release of a single cell from a trap, a PCB enabling selective addressing of electrodes is required. This PCB (Figure S2) was designed with KiCAD. The signal coming from the function generator is connected on the PCB through via an SMA connector and distributed over 5 8-fan-out multiplexers (ADG1414, Analog devices Inc. The forty outputs are connected singularly to spring-loaded contacts interfacing the PCB with the electrodes of the microfluidic chip. The configuration of the multiplexer is controlled by an Arduino system, which sends the command for closing or opening to the multiplexers through a Serial Peripheral Interface (SPI). The multiplexers are connected in daisy chain so that they can be addressed with a single bus. The holes in the PCB allow the mounting of the chip inside a metallic chip holder at the center of the PCB, and four screws are placed to ensure the contact between the pins and the pads. (**a-A**) CAD design of the PCB layout. Copper tracks are shown in red (top side) and green (bottom side). The tracks bringing the signal from the frequency generator to the multiplexers are red while the tracks bringing the signal from the multiplexers to the chip via spring-loaded contacts are depicted in green. The configuration of the multiplexers is set through an Arduino microcontroller. (**a-B**) Picture of the assembled PCB: power is supplied to the five multiplexers by the green connector at the top of the PCB. The electrical signal for DEP is provided by the SMA connector in the bottom center of the PCB. The Arduino controller visible at the bottom is connected to a computer through a micro-USB port. (**b**) **Python interface** developed to control the multiplexers’ configuration and allow independent addressing of each electrode in order to carry out selective retrieval of a single-cell. By clicking on the trap number, the program will send a signal to the Arduino that will subsequently change the multiplexer configuration to route the electrical signal to the trap of interest.

**
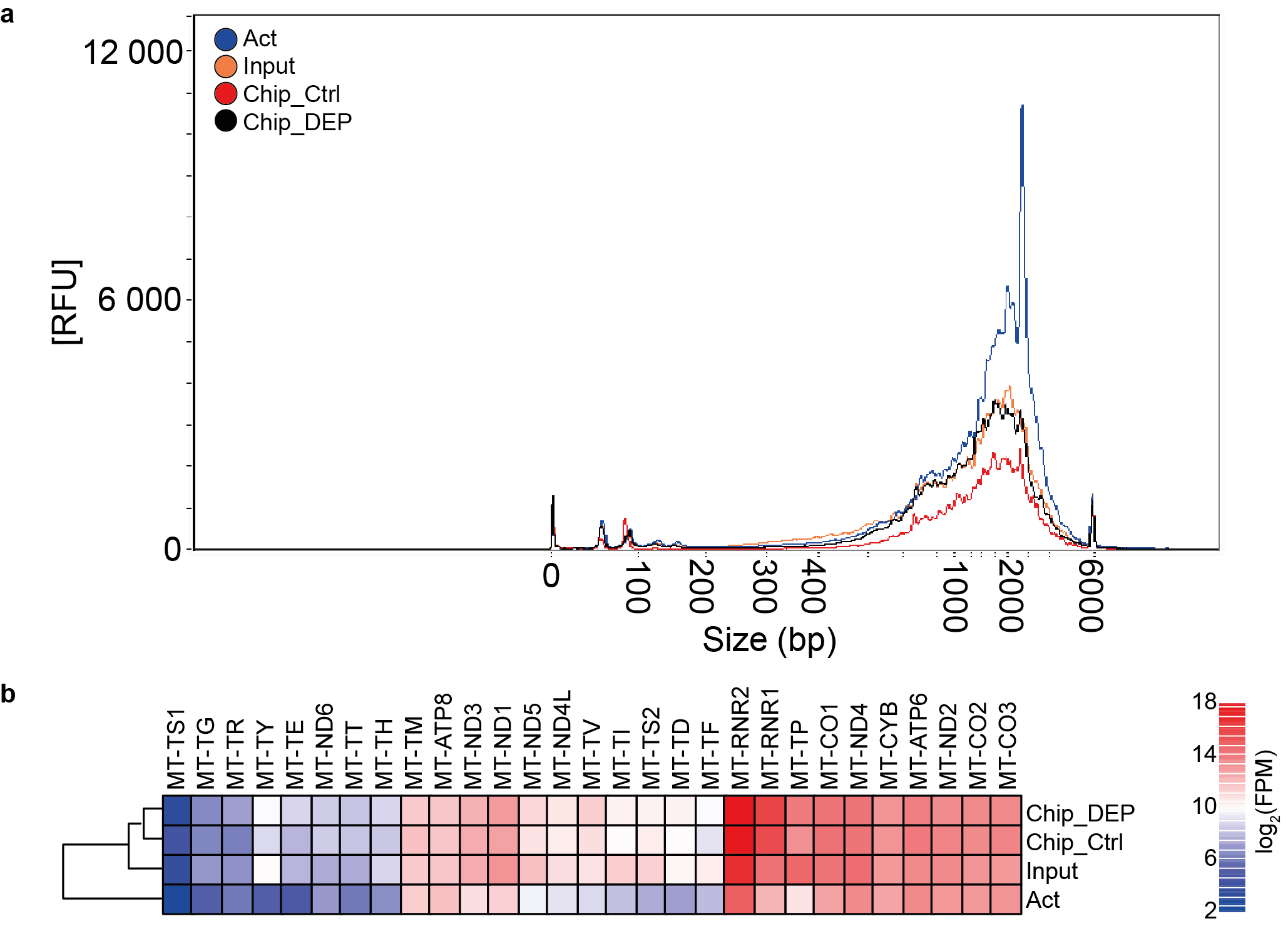
**

**Figure S4. Cell viability. MiPARC releases viable Jurkat T-cells.** Jurkat cells were either injected into the microfluidics chip (Chip-Ctrl) or additionally subjected to the electric field used for accurate capture and retrieval of cells (Chip-DEP). Controls were either the input cells (Input) or cells activated for three hours under Phorbol-12-myristate 13-acetate and Ionomcyin activation (Act). Cells from all conditions were cultured for three hours to permit transcriptional changes to take place subsequent to treatment. **a)** Fragment analyzer profile of cDNA libraries. **b)** Hierarchical clustering of expression of mitochondrial genes.
